# Effect of Efavirenz on UDP-Glucuronosyltransferase 1A1, 1A4, 1A6, and 1A9 Activities in Human Liver Microsomes

**DOI:** 10.3390/molecules17010851

**Published:** 2012-01-17

**Authors:** Hye Young Ji, Hyeri Lee, Sae Rom Lim, Jeong Han Kim, Hye Suk Lee

**Affiliations:** 1 National Research Laboratory of Drug Metabolism & Bioanalysis, College of Pharmacy, The Catholic University of Korea, Bucheon 420-743, Korea; 2 Department of Agricultural Biotechnology, Seoul National University, Seoul 151-742, Korea

**Keywords:** efavirenz, UDP-glucuronosyltransferase inhibition, human liver microsomes, drug-drug interaction

## Abstract

Efavirenz is a non-nucleoside reverse transcriptase inhibitor used for the treatment of human immunodeficiency virus type 1 infections. Drug interactions of efavirenz have been reported due to *in vitro* inhibition of CYP2C9, CYP2C19, CYP3A4, and UDP-glucuronosyltransferase 2B7 (UGT2B7) and *in vivo* CYP3A4 induction. The inhibitory potentials of efavirenz on the enzyme activities of four major UDP-glucuronosyltransferases (UGTs), 1A1, 1A4, 1A6, and 1A9, in human liver microsomes were investigated using liquid chromatography-tandem mass spectrometry. Efavirenz potently inhibited UGT1A4-mediated trifluoperazine *N*-glucuronidation and UGT1A9-mediated propofol glucuronidation, with *K*_i_ values of 2.0 and 9.4 μM, respectively. [I]/*K*_i_ ratios of efavirenz for trifluoperazine *N*-glucuronidation and propofol glucuronidation were 6.5 and 1.37, respectively. Efavirenz also moderately inhibited UGT1A1-mediated 17β-estradiol 3-glucuronidation, with a *K*_i_ value of 40.3 μM, but did not inhibit UGT1A6-mediated 1-naphthol glucuronidation. Those *in vitro* results suggest that efavirenz should be examined for potential pharmacokinetic drug interactions *in vivo* due to strong inhibition of UGT1A4 and UGT1A9.

## 1. Introduction

**Scheme 1:**
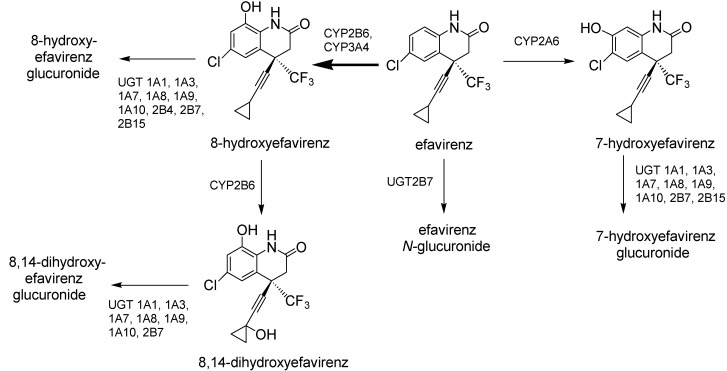
Metabolic pathways of efavirenz in humans.

Efavirenz is a non-nucleoside reverse transcriptase inhibitor (NNRTI) used in combination with other antiretroviral agents for the treatment of human immunodeficiency virus type 1 infections [1,2]. Metabolism studies of efavirenz in humans and *in vitro* studies have demonstrated that efavirenz is mainly metabolized to inactive 8-hydroxyefavirenz, 7-hydroxyefavirenz, and 8,14-dihydroxyefavirenz catalyzed by CYP2B6, CYP3A4, and CYP2A6, which are subsequently conjugated by multiple UDP-glucuronosyltransferases (UGTs) (Scheme 1) [3,4,5,6,7,8,9,10]. Formation of efavirenz *N*-glucuronide from efavirenz is catalyzed by UGT2B7 and may contribute minimally to the overall clearance of efavirenz [8,9,10].

*In vitro* studies have shown that efavirenz inhibited CYPs 2C9, 2C19, and 3A4, with *K*_i_ values (8.5 to 17 μM) in the range of observed efavirenz plasma concentrations, but efavirenz did not inhibit CYP2E1 and inhibited CYP2D6 and CYP 1A2 (*K*_i_ values 82 to 160 μM) only at concentrations well above those achieved clinically [3]. Efavirenz has been shown to cause hepatic enzyme induction *in vivo*, thus increasing the biotransformation of some drugs metabolized by CYP3A. Co-administration of efavirenz with drugs primarily metabolized by 2C9, 2C19, and 3A isozymes may result in altered plasma concentrations of the co-administered drug. Drugs that induce CYP3A activity would be expected to increase the clearance of efavirenz resulting in lowered plasma concentrations. Cisapride, midazolam, triazolam, ergot alkaloids and derivative (dihydroergotamine, ergonovine, ergotamine, and ergonovine), bepridil, pimozide, and St. John’s wort are contraindicated with efavirenz [3]. Efavirenz was shown to be one of the most selective and potent competitive inhibitors of UGT2B7-mediated azidothymidine glucuronidation, with a *K*_i_ value of 17 μM in human liver microsomes, supporting the idea that efavirenz could potentially interfere with azidothymidine glucuronidation *in vivo* [10].

UGT enzymes are divided into two families, UGT1 and UGT2, and three subfamilies, UGT1A (1A1, 1A3, 1A4, 1A5, 1A6, 1A7, 1A8, 1A9, and 1A10), 2A (2A1 and 2A2), and 2B (2B4, 2B7, 2B10, 2B11, 2B15, 2B17, and 2B28), based on sequence homology [11]. UGT enzymes are widely and differentially expressed throughout the human body, with the liver and as the main sites for xenobiotic glucuronidation [12,13]. Because many drugs and phytochemicals are glucuronidated by UGT1A1, UGT1A4, UGT1A6, and UGT1A9 enzymes, there is a potential for drug interaction through the modulation of those UGT enzyme activities [14,15,16,17]. Selective probes for the evaluation of UGT1A1, UGT1A4, UGT1A6, and UGT1A9 activities in UGT inhibition studies are also available [15,18,19,20].

To our knowledge, no previous study has reported the effect of efavirenz on other human UGT enzymes except UGT2B7. In this study, the effect of efavirenz on the activities of four major human UGTs, 1A1, 1A4, 1A6, and 1A9, were examined using pooled human liver microsomes to evaluate the possibility of efavirenz-drug interactions due to the inhibition of UGTs.

## 2. Results and Discussion

**Figure 1:**
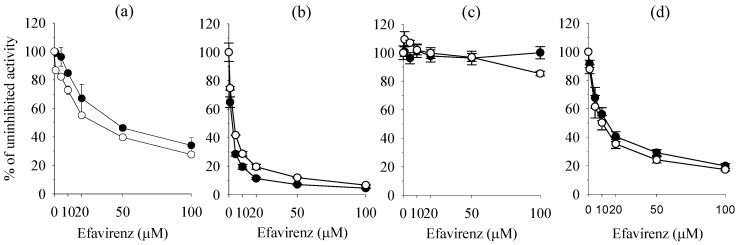
Inhibitory effect of efavirenz on (**a**) UGT1A1-catalyzed 17β-estradiol 3-glucuronidation; (**b**) UGT1A4-catalyzed trifluoperazine *N*-glucuronidation; (**c**) UGT1A6-catalyzed naphthol 1-glucuronidation; and (**d**) UGT1A9-catalyzed propofol glucuronidation in pooled human liver microsomes H161 (●) and each human cDNA-expressed UGT1A1, 1A4, 1A6, and 1A9 supersomes (○).

**Figure 2:**
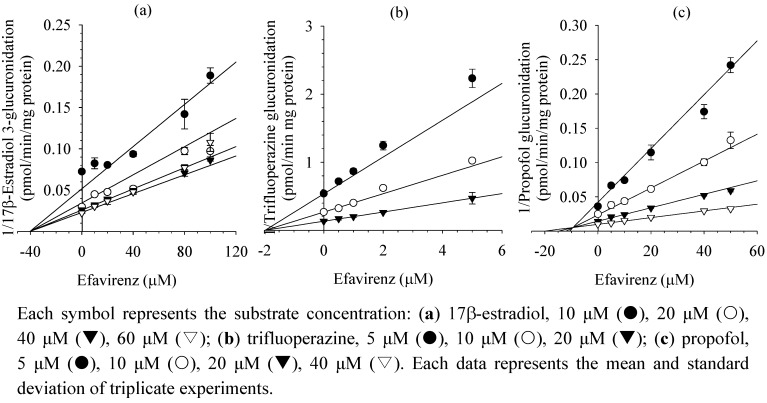
Representative Dixon plots for inhibitory effects of efavirenz on (**a**) UGT1A1-catalyzed 17β-estradiol 3-glucuronidation; (**b**) UGT1A4-catalyzed trifluoperazine *N*-glucuronidation; and (**c**) UGT1A9-catalyzed propofol glucuronidation in pooled human liver microsomes H161.

The inhibitory effects of efavirenz on four major human UGT enzymes, 1A1, 1A4, 1A6, and 1A9, were evaluated using each UGT probe substrate in human liver microsomes and human cDNA-expressed UGT isozymes. IC_50_ values of efavirenz inhibited UGT1A1-mediated 17β-estradiol 3-glucuronidation, UGT1A4-mediated trifluoperazine *N*-glucuronidation, and UGT1A9-mediated propofol glucuronidation, were 45.9, 2.1, and 15.8 μM, respectively, in human liver microsomes and 33.8, 4.0, and 11.6 μM, respectively, in each UGT isozyme (Table 1, Figure 1). Efavirenz at 100 μM showed negligible inhibition of UGT1A6-mediated 1-naphthol glucuronidation in human liver microsomes and UGT1A6 isozyme. Efavirenz showed noncompetitive inhibition for 17β-estradiol 3-glucuronidation, with a *K*_i _value of 40.3 μM, which was higher than the steady-state maximum plasma concentrations of efavirenz (12.9 μM) [22], suggesting that drug interaction of efavirenz based on UGT1A1 inhibition is not possible. Efavirenz competitively inhibited trifluoperazine *N*-glucuronidation and propofol glucuronidation, with *K*_i_ values of 2.0 and 9.4 μM, respectively, in human liver microsomes (Table 1 and Figure 2).

**Table 1:** Effect of efavirenz on UGT metabolic activity in pooled human liver microsomes H161.

UGT	Marker enzyme	IC_50_ (μM)	*K*_i_ (μM)	Inhibition mode
UGT1A1	17β-Estradiol-3-glucuronidation	45.9 ± 6.4	40.3 ± 0.6	Noncompetitive
UGT1A4	Trifluoperazine *N-*glucuronidation	2.1 ± 0.2	2.0 ± 0.3	Competitive
UGT1A6	Naphthol 1-glucuronidation	No Inhibition	-	-
UGT1A9	Propofol glucuronidation	15.8 ± 2.8	9.4 ± 0.9	Competitive

Efavirenz has shown potent inhibitory activity of trifluoperazine *N*-glucuronidation similar to a selective UGT1A4 inhibitor, hecogenine (IC_50_, 1.5 μM) [18]. After an efavirenz oral dose of 600 mg daily, steady-state maximum plasma concentration (C_max_) and minimum plasma concentration (C_min_) values of efavirenz are 12.9 and 5.6 μM, respectively, with a half-life of more than 40 hours [22]. Considering that the ratio of steady-state C_max_ of efavirenz to its apparent *K*_i_ (2.0 μM) ([I]/*K*_i_) is 6.5, the inhibition of efavirenz on UGT1A4-mediated trifluoperazine *N*-glucuronidation is likely, but remains to be demonstrated *in vivo*. According to this *in vitro* data, efavirenz should be used carefully with the drugs metabolized by UGT1A4, such as antifungal drugs (alprazolam, posaconazole, ketoconazole, miconazole) [23], hydroxymidazolam [24], tamoxifen [25], lamotrigine [26], and tacrolimus [27], in order to avoid drug interactions.

The *K*_i_ value (9.4 μM) for inhibition of efavirenz on UGT1A9-mediated propofol glucuronidation was higher than those produced by potent inhibitors of UGT1A9, niflumic acid (*K*_i_, 0.1–0.4 μM) [28,29] and sorafenib (*K*_i_, 2 μM) [30] but was in the range of its steady-state C_max_ and C_min_ values. Since the [I]/*K*_i_ ratio of efavirenz for UGT1A9-mediated propofol glucuronidation was 1.37, inhibition of glucuronidation of UGT1A9 substrates by efavirenz may be possible, suggesting that efavirenz may be used carefully with drugs metabolized by UGT1A9, such as *S*-etodolac [31], entacapone [32], gaboxadol [33], retigabine [34], and scopoletin [35], to avoid drug interactions.

Bélanger *et al.* [10] estimated that efavirenz, a selective substrate of UGT2B7, with *K*_i_ value of 17 μM, could reduce azidothymidine glucuronidation by approximately 43% at steady-state C_max_ vlaues. *K*_i_ values of efavirenz for inhibition of UGT1A4-mediated trifluoperazine *N*-glucuronidation and UGT1A9-mediated propofol glucuronidation were 2.0 and 9.4 μM, respectively, which were much less than its *K*_i_ for UGT2B7-mediated azidothymidine glucuronidation (17.4 μM in our unpublished data). Those *in vitro* results indicate that efavirenz can potentially inhibit the glucuronidation of drugs catalyzed by UGT1A4 and/or UGT1A9 and therefore should be examined for potential pharmacokinetic drug interactions *in vivo* due to inhibition of UGT1A4 and UGT1A9.

## 3. Experimental

### 3.1. Materials and Reagents

Efavirenz and propofol glucuronide were obtained from Toronto Research Chemicals (Toronto, ON, Canada). 17β-Estradiol, 17β-estradiol 3-glucuronide, 1-naphthol, naphthol glucuronide, propofol, trifluoperazine, alamethicin (from *Trichoderma viride*), hecogenin, and uridine-5-diphosphoglucuronic acid trisodium salt (UDPGA) were obtained from Sigma-Aldrich (St. Louis, MO, USA). Pooled human liver microsomes (H161) were obtained from BD Gentest Co. (Woburn, MA, USA). Acetonitrile and methanol (HPLC grade) were obtained from Burdick & Jackson Inc. (Muskegon, MI, USA), and the other chemicals were of the highest quality available.

### 3.2. Inhibitory Effects of Efavirenz on Activities of Four UGTs in Human Liver Microsomes

The inhibitory potencies (IC_50_ values) of efavirenz were determined with UGT assays in the presence and absence of efavirenz (final concentrations of 0–200 μM with acetonitrile concentration less than 0.5% *v/v*) using pooled human liver microsomes and human cDNA-expressed UGT1A1, UGT1A4, UGT1A6 and UGT1A9 isozymes. The incubation mixtures were prepared in a total volume of 100 μL as follows: pooled human liver microsomes or UGT isozymes (0.2 mg/mL for 17β-estradiol and trifluoperazine; 0.1 mg/mL for 1-naphthol and propofol), 2 mM UDPGA, 25 μg/mL alamethicin, 10 mM MgCl_2_, 50 mM tris buffer (pH 7.4), UGT-isoform specific probe substrate (20 μM 17β-estradiol for UGT1A1, 5 μM for trifluoperazine for UGT1A4, 20 μM 1-naphthol for UGT1A6, and 10 μM propofol for UGT1A9), and various concentrations of efavirenz (0–200 μM). Reactions were initiated by the addition of UDPGA, and incubations were carried out at 37 °C in a shaking water bath for 30 min. Reactions were terminated by addition of 100 μL of ice-cold methanol containing internal standard (3 μg/mL ezetimibe for 17β-estradiol 3-glucuronide, 1-naphthol glucuronide, and propofol glucuronide; 0.3 μg/mL meloxicam for trifluoperazine *N*-glucuronide). The incubation mixtures were centrifuged at 13,000 × g for 5 min, and then 40 μL of the supernatant was diluted with 60 μL of water. The aliquot (5 μL) was injected onto an LC/MS/MS instrument. All incubations were performed in triplicate and the mean values were used. The glucuronides produced from UGT isoform-specific substrates were respectively determined by LC/MS/MS [21]. The system consisted of a tandem quadrupole mass spectrometer (TSQ Quantum Access, ThermoFisher Scientific, San Jose, CA, USA) coupled with a Nanospace SI-2 LC system (Shiseido, Tokyo, Japan). The separation was performed on an Atlantis dC18 column (5 μm, 2.1 mm i.d. × 100 mm, Waters, MA, USA) using the gradient elution of a mixture of 5% methanol in 0.1% formic acid (mobile phase A) and 95% methanol in 0.1% formic acid (mobile phase B) at a flow rate of 0.25 mL/min: 10% mobile phase B for 2 min and 10% to 95% mobile phase B for 4 min. The column and autosampler temperatures were 50 and 6 °C, respectively. After 3.0 min, the LC eluent was diverted from waste to the mass spectrometer fitted with electrospray ionization (ESI) source. The ESI source settings were as following: ESI voltage for trifluoperazine *N*-glucuronide, +5.0 kV; electrospray voltage for 17β-estradiol 3-glucuronide, 1-naphthol glucuronide, and propofol glucuronide, −4.0 kV; vaporizer temperature, 420 °C; capillary temperature 360 °C; sheath gas pressure, 35 psi; and auxiliary gas pressure, 10 psi. Quantification was performed by selected reaction monitoring (SRM) and SRM transitions for the metabolites are summarized in Table 2. The analytical data were processed by Xcalibur^®^ software (Thermo Fisher Scientific).

**Table 2:** LC/MS/MS measurement conditions for drug glucuronidation catalyzed by human UGT enzymes.

Enzymes	Compound	Polarity	SRM Transition	Tube lens (V)	Collision energy (V)
*Metabolite*					
UGT1A1	17β-Estradiol-3-glucuronide	negative	446.9 > 270.9	94	34
UGT1A4	Trifluoperazine *N*-glucuronide	positive	584.20 > 408.13	94	27
UGT1A6	Naphthol 1-glucuronide	negative	319.48 > 143.30	72	18
UGT1A9	Propofol glucuronide	negative	353.18 > 177.19	63	20
*Internal standard*					
UGT 1A1, 1A6, 1A9	Ezetimibe	negative	408.07 > 271.43	45	21
UGT 1A4	Meloxicam	positive	352.05 > 115.38	63	20

*K*_i_ values for UGT1A1, UGT1A4, and UGT1A9 in human liver microsomes were determined after the enzymes were incubated with various concentrations of substrates (10–60 μM 17β-estradiol for UGT1A1, 5–20 μM trifluoperazine for UGT1A4, and 5–40 μM propofol for UGT1A9), 2 mM UDPGA, 25 μg/mL alamethicin, 10 mM MgCl_2_, and various concentrations of efavirenz in 50 mM Tris buffer (pH 7.4) in a total incubation volume of 100 μL. Reactions were initiated by addition of UDPGA at 37 °C and stopped after 30 min by placement of the incubation tubes on ice and addition of 100 μL ice-cold methanol containing an internal standard described above. The incubation mixtures were centrifuged at 13,000 × g for 5 min, followed by dilution of 40 μL of the supernatant with 60 μL of water. The aliquot (5 μL) was analyzed by LC/MS/MS.

### 3.3. Data Analysis

The IC_50_ values (concentration of inhibitor causing 50% inhibition of the original enzyme activity) were calculated using WinNonlin software, a non-linear regression analysis program (Pharsight, Mountain View, CA, USA). The apparent kinetic parameters for inhibitory potential (*K*_i_ values) were estimated from the fitted curves using Enzyme Kinetics Ver. 1.3 program (Systat Software Inc., San Jose, CA, USA).

## 4. Conclusions

The effect of efavirenz on four UGTs, 1A1, 1A4, 1A6, and 1A9, was evaluated across a wide range of substrate and efavirenz concentrations using *in vitro* human liver microsomes. UGT1A4-mediated trifluoperazine *N*-glucuronidation and UGT1A9-mediated propofol glucuronidation activities were potently inhibited by efavirenz during incubation with UDPGA in microsomes. Efavirenz also inhibited UGT1A1-mediated 17β-estradiol 3-glucuronidation in a dose-dependent manner but did not inhibit UGT1A6-mediated 1-naphthol glucuronidation. Those results suggest that efavirenz has the potential to cause pharmacokinetic drug interactions with other co-administered drugs metabolized by UGT1A4 and UGT1A9. However, the clinical relevance of the inhibitory interaction of efavirenz with UGT1A4- and UGT1A9-substrate drugs has not been investigated. Clinical trials to evaluate the inhibitory effects of efavirenz on UGT1A4 and UGT1A9 remain to be conducted.

## References

[1] Rakhmanina N.Y., van den Anker J.N. (2010). Efavirenz in the therapy of HIV infection. Expert Opin. Drug Metab. Toxicol..

[2] Deeks E.D., Perry C.M. (2010). Efavirenz/emtricitabine/tenofovir disoproxil fumarate single-tablet regimen (Atripla(R)): A review of its use in the management of HIV infection. Drugs.

[3] U.S. Prescribing Information. http://packageinserts.bms.com/pi/pi_sustiva.pdf.

[4] Mutlib A.E., Chen H., Nemeth G.A., Markwalder J.A., Seitz S.P., Gan L.S., Christ D.D. (1999). Identification and characterization of efavirenz metabolites by liquid chromatography/mass spectrometry and high field NMR: Species differences in the metabolism of efavirenz. Drug Metab. Dispos..

[5] Ward B.A., Gorski J.C., Jones D.R., Hall S.D., Flockhart D.A., Desta Z. (2003). The cytochrome P450 2B6 (CYP2B6) is the main catalyst of efavirenz primary and secondary metabolism: Implication for HIV/AIDS therapy and utility of efavirenz as a substrate marker of CYP2B6 catalytic activity. J. Pharmacol. Exp. Ther..

[6] Ogburn E.T., Jones D.R., Masters A.R., Xu C., Guo Y., Desta Z. (2010). Efavirenz primary and secondary metabolism *in vitro* and *in vivo*: Identification of novel metabolic pathways and cytochrome P450 2A6 as the principal catalyst of efavirenz 7-hydroxylation. Drug Metab. Dispos..

[7] Rekic D., Roshammar D., Mukonzo J., Ashton M. (2011). In silico prediction of efavirenz and rifampicin drug-drug interaction considering weight and CYP2B6 phenotype. Br. J. Clin. Pharmacol..

[8] Bae S.K., Jeong Y.J., Lee C., Liu K.H. (2011). Identification of human UGT isoforms responsible for glucuronidation of efavirenz and its three hydroxy metabolites. Xenobiotica.

[9] Cho D.Y., Ogburn E.T., Jones D., Desta Z. (2011). Contribution of N-glucuronidation to efavirenz elimination *in vivo* in the basal and rifampin-induced metabolism of efavirenz. Antimicrob. Agents Chemother..

[10] Belanger A.S., Caron P., Harvey M., Zimmerman P.A., Mehlotra R.K., Guillemette C. (2009). Glucuronidation of the antiretroviral drug efavirenz by UGT2B7 and an *in vitro* investigation of drug-drug interaction with zidovudine. Drug Metab. Dispos..

[11] Guillemette C., Levesque E., Harvey M., Bellemare J., Menard V. (2010). UGT genomic diversity: Beyond gene duplication. Drug Metab. Rev..

[12] Izukawa T., Nakajima M., Fujiwara R., Yamanaka H., Fukami T., Takamiya M., Aoki Y., Ikushiro S., Sakaki T., Yokoi T. (2009). Quantitative analysis of UDP-glucuronosyl transferase (UGT) 1A and UGT2B expression levels in human livers. Drug Metab. Dispos..

[13] Ohno S., Nakajin S. (2009). Determination of mRNA expression of human UDP-glucuronosyl transferases and application for localization in various human tissues by real-time reverse transcriptase-polymerase chain reaction. Drug Metab. Dispos..

[14] Kiang T.K., Ensom M.H., Chang T.K. (2005). UDP-glucuronosyltransferases and clinical drug-drug interactions. Pharmacol. Ther..

[15] Mohamed M.F., Frye R.F. (2011). Effects of herbal supplements on drug glucuronidation. Review of clinical, animal, and *in vitro* studies. Planta Med..

[16] Ebert U., Thong N.Q., Qertel R., Kirch W. (2000). Effects of rifampicin and cimetidine on pharmacokinetics and pharmacodynamics of lamotrigine in healthy subjects. Eur. J. Clin. Pharmacol..

[17] van der Lee M.J., Dawood I., ter Hofstede H.J., de Graaff-Teulen M.J., van Ewijk-Beneken Kolmer E.W., Caliskan-Yassen N., Koopmans P.P., Burger D.M. (2006). Lopinavir/ritonavir reduces lamotrigine plasma concentrations in healthy subjects. Clin. Pharmacol. Ther..

[18] Uchaipichat V., Mackenzie P.I., Elliot D.J., Miners J.O. (2006). Selectivity of substrate (trifluoperazine) and inhibitor (amitriptyline, androsterone, canrenoic acid, hecogenin, phenylbutazone, quinidine, quinine, and sulfinpyrazone) “probes” for human UDP-glucuronosyltransferases. Drug Metab. Dispos..

[19] Court M.H. (2005). Isoform-selective probe substrates for *in vitro* studies of human UDP-glucuronosyltransferases. Methods Enzymol..

[20] Donato M.T., Montero S., Castell J.V., Gómez-Lechón M.J., Lahoz A. (2010). Validated assay for studying activity profiles of human liver UGTs after drug exposure: Inhibition and induction studies. Anal. Bioanal. Chem..

[21] Ji H.Y., Liu K.H., Lee H., Im S.R., Shim H.J., Son M., Lee H.S. (2011). Corydaline inhibits multiple cytochrome P450 and UDP-glucuronosyltransferase enzyme activities in human liver microsomes. Molecules.

[22] Marzolini C., Telenti A., Decosterd L.A., Greub G., Biollaz J., Buclin T. (2001). Efavirenz plasma levels can predict treatment failure and central nervous system side effects in HIV-1-infected patients. AIDS.

[23] Bourcier K., Hyland R., Kempshall S., Jones R., Maximilien J., Irvine N., Jones B. (2010). Investigation into UDP-glucuronosyltransferase (UGT) enzyme kinetics of imidazole- and triazole-containing antifungal drugs in human liver microsomes and recombinant UGT enzymes. Drug Metab. Dispos..

[24] Hyland R., Osborne T., Payne A., Kempshall S., Logan Y.R., Ezzeddine K., Jones B. (2009). *In vitro* and *in vivo* glucuronidation of midazolam in humans. Br. J. Clin. Pharmacol..

[25] Kaku T., Ogura K., Nishiyama T., Ohnuma T., Muro K., Hiratsuka A. (2004). Quaternary ammonium-linked glucuronidation of tamoxifen by human liver microsomes and UDP-glucuronosyltransferase 1A4. Biochem. Pharmacol..

[26] Rowland A., Elliot D.J., Williams J.A., Mackenzie P.I., Dickinson R.G., Miners J.O. (2006). *In vitro* characterization of lamotrigine N2-glucuronidation and the lamotrigine-valproic acid interaction. Drug Metab. Dispos..

[27] Laverdiere I., Caron P., Harvey M., Levesque E., Guillemette C. (2011). *In vitro* investigation of human UDP-glucuronosyltransferase isoforms responsible for tacrolimus glucuronidation: Predominant contribution of UGT1A4. Drug Metab. Dispos..

[28] Miners J.O., Bowalgaha K., Elliot D.J., Baranczewski P., Knights K.M. (2011). Characterization of niflumic acid as a selective inhibitor of human liver microsomal UDP-glucuronosyltransferase 1A9: Application to the reaction phenotyping of acetaminophen glucuronidation. Drug Metab. Dispos..

[29] Mano Y., Usui T., Kamimura H. (2006). *In vitro* inhibitory effects of non-steroidal anti-inflammatory drugs on 4-methylumbelliferone glucuronidation in recombinant human UDP-glucuronosyltransferase 1A9-potent inhibition by niflumic acid. Biopharm. Drug Dispos..

[30] Nexavar (Sorafenib) - Drug Interactions, Contraindications, Overdosage. http://www.druglib.com/druginfo/nexavar/interactions_overdosage_contraindications/.

[31] Tougou K., Gotou H., Ohno Y., Nakamura A. (2004). Stereoselective glucuronidation and hydroxylation of etodolac by UGT1A9 and CYP2C9 in man. Xenobiotica.

[32] Lautala P., Ethell B.T., Taskinen J., Burchell B. (2000). The specificity of glucuronidation of entacapone and tolcapone by recombinant human UDP-glucuronosyltransferases. Drug Metab. Dispos..

[33] Chu X.Y., Liang Y., Cai X., Cuevas-Licea K., Rippley R.K., Kassahun K., Shou M., Braun M.P., Doss G.A., Anari M.R. (2009). Metabolism and renal elimination of gaboxadol in humans: Role of UDP-glucuronosyltransferases and transporters. Pharm. Res..

[34] Borlak J., Gasparic A., Locher M., Schupke H., Hermann R. (2006). *N*-Glucuronidation of the antiepileptic drug retigabine: Results from studies with human volunteers, heterologously expressed human UGTs, human liver, kidney, and liver microsomal membranes of Crigler-Najjar type II. Metabolism.

[35] Luukkanen L., Taskinen J., Kurkela M., Kostiainen R., Hirvonen J., Finel M. (2005). Kinetic characterization of the 1A subfamily of recombinant human UDP-glucuronosyltransferases. Drug Metab. Dispos..

